# Thrombin Induces Macrophage Migration Inhibitory Factor Release and Upregulation in Urothelium: A Possible Contribution to Bladder Inflammation

**DOI:** 10.1371/journal.pone.0015904

**Published:** 2010-12-31

**Authors:** Pedro L. Vera, Terra E. Wolfe, Alexander E. Braley, Katherine L. Meyer-Siegler

**Affiliations:** 1 Research and Development, The Bay Pines VA Healthcare System, Bay Pines, Florida, United States of America; 2 Division of Urology, Department of Surgery, University of South Florida, Tampa, Florida, United States of America; 3 Department of Molecular Medicine, University of South Florida, Tampa, Florida, United States of America; Fundação Oswaldo Cruz, Brazil

## Abstract

**Purpose:**

Macrophage migration inhibitory factor (MIF) is a pro-inflammatory cytokine expressed by urothelial cells that mediates bladder inflammation. We investigated the effect of stimulation with thrombin, a Protease Activated Receptor-1 (PAR1) agonist, on MIF release and MIF mRNA upregulation in urothelial cells.

**Materials and Methods:**

MIF and PAR1 expression was examined in normal human immortalized urothelial cells (UROtsa) using real-time RT-PCR, Western blotting and dual immunostaining. MIF and PAR1 immunostaining was also examined in rat urothelium. The effect of thrombin stimulation (100 nM) on urothelial MIF release was examined in UROtsa cells (*in vitro*) and in rats (*in vivo*). UROtsa cells were stimulated with thrombin, culture media were collected at different time points and MIF amounts were determined by ELISA. Pentobarbital anesthetized rats received intravesical saline (control), thrombin, or thrombin +2% lidocaine (to block nerve activity) for 1 hr, intraluminal fluid was collected and MIF amounts determined by ELISA. Bladder or UROtsa MIF mRNA was measured using real time RT-PCR.

**Results:**

UROtsa cells constitutively express MIF and PAR1 and immunostaining for both was observed in these cells and in the basal and intermediate layers of rat urothelium. Thrombin stimulation of urothelial cells resulted in a concentration- and time-dependent increase in MIF release both *in vitro* (UROtsa; 2.8-fold increase at 1 hr) and *in vivo* (rat; 4.5-fold) while heat-inactivated thrombin had no effect. In rats, thrombin-induced MIF release was reduced but not abolished by intravesical lidocaine treatment. Thrombin also upregulated MIF mRNA in UROtsa cells (3.3-fold increase) and in the rat bladder (2-fold increase) where the effect was reduced (1.4-fold) by lidocaine treatment.

**Conclusions:**

Urothelial cells express both MIF and PAR1. Activation of urothelial PAR1 receptors, either by locally generated thrombin or proteases present in the urine, may mediate bladder inflammation by inducing urothelial MIF release and upregulating urothelial MIF expression.

## Introduction

Macrophage migration inhibitory (MIF), the earliest identified cyokine, was originally described as produced by activated T cells and capable of stopping the random migration of macrophages *in vitro* [1], [2]. Presently, MIF is recognized as a pleiotropic cytokine that functions as a pivotal mediator of acute and chronic inflammation and is synthesized by a variety of cell types and organs [3]–[5].

MIF is constitutively expressed by urothelial cells and mediates inflammation in the bladder [6], [7]. Inflammatory stimuli elicit MIF release from the urothelium into the bladder lumen and upregulation of MIF expression by the bladder in general and urothelium in particular [8], [9]. Released luminal MIF binds and activates receptors for MIF expressed by urothelial cells [10], [11] to induce a cascade of other inflammatory cytokines to be produced by the bladder and urothelium [8], [9]. Therefore, release of urothelial preformed MIF and activation of MIF production in the bladder (and urothelium in particular) by inflammatory stimuli are key elements in MIF-mediated bladder inflammation. Understanding the triggers evoking urothelial MIF release is an important component of understanding how cystitis is developed or maintained.

Protease activated receptors (PAR) are a unique class of receptors that carry their own ligands tethered to the receptor complex. Proteases clip and free the tethered ligand to bind to the receptor and mediate signal transduction [12], [13]. To date, four different PAR receptors have been identified and they are implicated in mediating inflammation and pain, among other functions [12]–[14]. Thrombin is a serine protease with high affinity for PAR1 and much lower affinity for PAR4 receptors [12], [15]. Recently, thrombin was shown to elicit MIF release and MIF mRNA upregulation from human endothelial cells *in vitro* [16], [17]. Moreover, a specific PAR1 agonist also elicited MIF mRNA upregulation establishing that thrombin-mediated MIF effects are due to its well-described affinity for PAR1 receptors [16].

PAR receptors, although not studied in extensive detail in the urogenital tract [18], have been described in primary human urothelial cells and urothelial cancer cells *in vitro* [19]–[21]. In addition, PAR1-4 receptors were described in mouse urothelium [22], while only PAR2-4 receptors have been examined in the rat bladder [23]. Given that urothelial cells express PAR1 receptors and also constitutively synthesize MIF and release MIF (as reviewed above) in response to inflammatory stimuli, we hypothesized that thrombin would elicit MIF release from urothelial cells. Therefore, as part of our investigation of MIF-mediated bladder inflammation, we examined whether: 1) Transformed normal human urothelial cells expressed PAR receptors in general, and PAR1 receptor specifically and also whether they express MIF; 2) the location of PAR1 receptors (since it had not been described) and MIF in rat urothelium; 3) whether thrombin stimulation elicits MIF release from human urothelial cells *in vitro* and from rat urothelial cells *in vivo* and 4) whether thrombin stimulation elicits MIF upregulation in human urothelial cells *in vitro* and from rat urothelial cells *in vivo*.

## Results

### UROtsa cells express MIF and PAR receptors

**Figure 1:**
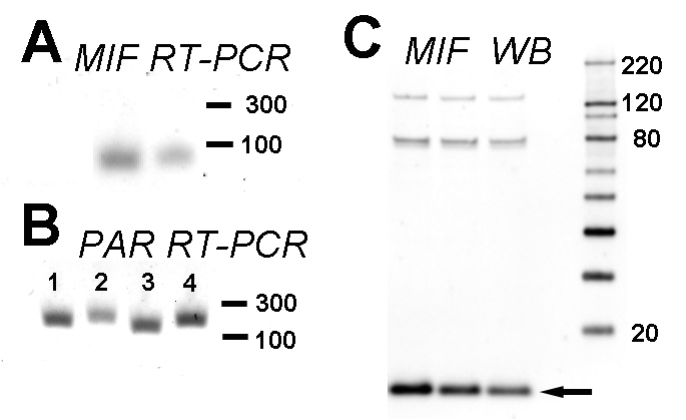
UROtsa cell constitutively express MIF and PAR receptors. Results from RT-PCR experiments showed that UROtsa cells express MIF mRNA (A) and all 4 PAR receptors (B; lanes 1–4 represent PAR1-4 respectively). MIF Western-blotting of UROtsa homogenates (3 representative samples included; C) showed a strong band at approximately 12 kDa corresponding to monomeric MIF (arrow). In addition, 2 distinct MIF bands were observed at higher molecular weight (approximately 80 and 120 kDa) corresponding to MIF binding to protein complexes as described in other systems [24], [25].

Since MIF and/or PAR receptors had not been studied in UROtsa cells, we examined expression of these two proteins in UROtsa cells. RT-PCR showed that UROtsa cells constitutively expressed MIF (Fig. 1A) and PAR receptors 1 through 4 (Fig. 1B). In addition, MIF was identified in UROtsa cell lysates using western blotting (Fig. 1C). A strong band at 12 kDa was identified, corresponding to monomeric MIF (Fig. 1C; arrow). Two prominent higher molecular weight MIF bands were also detected at approximately 80 and 120 kDa (Fig. 1C) corresponding to MIF-complexes, as described in other systems [24], [25].

### PAR1 and MIF immunostaining in urothelium

We tested three different PAR1 antibodies for their ability to detect PAR1 in formaldehyde-fixed rat bladder sections and compared the results to snap-frozen bladder cryostat sections that were post-fixed with acetone-PBS. We also compared the effects of formaldehyde fixation vs acetone-PBS fixation on PAR1 immunostaining in UROtsa cells. Of the three different antibodies tested, only one (Goat anti-PAR1; R&D Systems) gave positive results and only in acetone-fixed tissue. Consistently, formaldehyde fixation abolished PAR1 immunostaining in rat bladder when compared to acetone fixation. Formaldehyde fixation also reduced PAR immunostaining intensity in rat dorsal root ganglia [23]. UROtsa cells immunostaining was not diminished by formaldehyde fixation.

**Figure 2:**
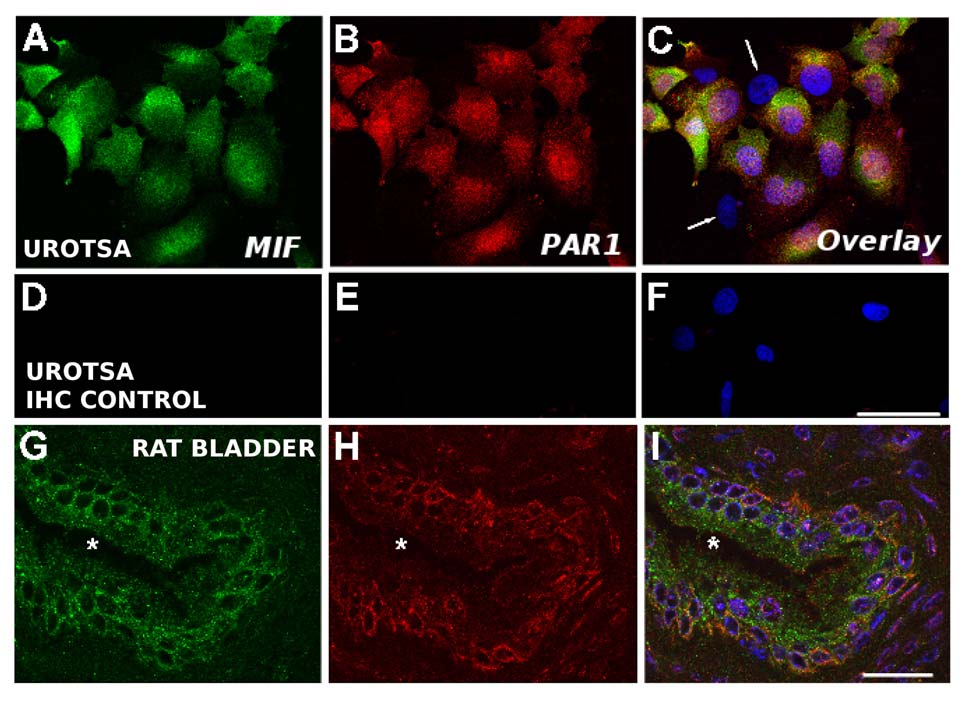
MIF and PAR1 immunostaining in urothelial cells. Representative samples from MIF, PAR1 immunostaining and an overlay showing both and nuclear staining (DAPI; blue). UROtsa cells displayed MIF (A) and PAR1 (B) immunostaining simultaneously in the same cell (C; overlay). However, a number of cells were observed that displayed neither immunostaining (C; arrows) and only showed nuclear staining. Control slides where primary antisera had been omitted (D;E) showed only nuclear staining (F). In rat urothelium, MIF immunostaining was detected in basal and intermediate cells with surface cells displaying weak or no MIF immunofluorescence. PAR1 immunostaining was also observed in rat urothelium, mainly on basal cells but also in some intermediate cells (H) while surface cells showed no PAR1 staining. Overlay of the single staining panels showed that basal cells and some intermediate cells were positive for both MIF and PAR1. Calibration bar = 20 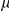
m.

We examined PAR1 and MIF immunostaining in UROtsa cells and rat urothelium using scanning laser confocal dual immunofluorescence. UROtsa cells showed MIF and PAR1 immunostaining. Figure 2 shows a representative field of UROtsa cells displaying MIF (Fig. 2A) and PAR1 (Fig. 2B) immunostaining individually and an overlay of the two fields (Fig. 2C). PAR1 immunostaining was located mostly on the cell surface, although cytoplasmic staining was also noted (Fig. 2B). Cells that showed PAR1 immunostaining also displayed MIF immunostaining (Fig. 2C). However, considerable heterogeneity of PAR1 immunostaining was observed with some cells showing intense PAR1 immunostaining while other cells were devoid of PAR1 or MIF immunostaining (Fig. 2C; arrows). The percent of unlabeled (showing neither MIF nor PAR1 immunostaining) UROtsa cells (determined by counting unlabeled cells in 20 separate fields at 20x magnification in two separate experiments) was 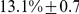 (S.E.M.). Immunohistochemistry control slides where primary antisera had been omitted showed no immunofluorescence (except for nuclear staining with DAPI; Fig. 2D–F). Also, slides where one of the primary antisera was omitted showed only immunofluorescence appropriate in the appropriate wavelength (not shown).

In rat urothelium, basal and intermediate cells showed MIF immunostaining, while superficial cells were lightly immunostained or devoid of MIF immunostaining (Fig. 2G) as reported earlier [7]. Co-immunostaining with PAR1 revealed immunofluorescence also in basal cells, where it appeared stronger, and in intermediate cells but not superficial cells (Fig. 2H). Similar to results in UROtsa cells, PAR1 staining was not homogeneous and areas of relatively intense staining were interspersed with areas of weaker PAR1 immunostaining. Composite images showed that PAR1 and MIF immunostaining was limited to basal and occasional intermediate cells (Fig. 2I). Sections where primary antisera had been omitted showed no immunofluorescence (not shown) and omission of either one of the primary antibodies resulted in fluorescence only in the appropriate wavelength (not shown).

### Thrombin evokes MIF release and upregulates MIF mRNA UROtsa cells

**Figure 3:**
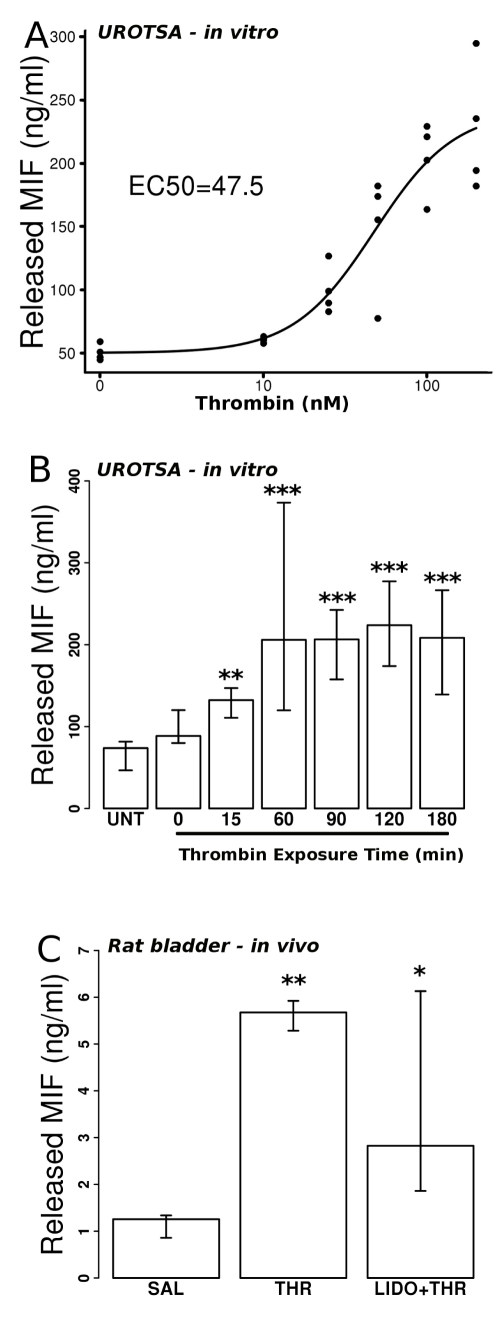
Thrombin elicits MIF release from urothelial cells. A) Effect of increasing concentration of thrombin (nM) on MIF (ng/ml) released into the culture medium and measured by ELISA. Cells treated with equivalent volume of sterile saline (0 nM thrombin) served as control. EC50 was 47.5 nM thrombin (calculated using a log-logistic regression [42]). B) MIF amounts (ng/ml) detected in culture media of UROtsa cells that were stimulated with thrombin (100 nM). Untreated cells (UNT) served as controls. Significant increases in the amount of MIF in the culture media were observed as early as 15 min after thrombin treatment and this effect peaked at 60 min and remained elevated throughout the entire time of the experiment (180 min). C) MIF amounts were assayed from rat intraluminal fluid collected after 1 hr of intravesical thrombin (100 nM) stimulation. Intravesical thrombin treatment (THR; n = 6) significantly increased the amounts of MIF present in the intraluminal fluid compared to intravesical saline treatment (SAL; n = 6). This effect was reduced, but not abolished by simultaneous intravesical treatment with 2% lidocaine and thrombin (LIDO+THR; n = 6). ANOVA determined overall significance while Dunnet's tests, using either the Untreated (UNT) group or saline (SAL) group as controls for UROtsa and rat experiments respectively. * = 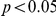; ** = 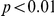; *** = 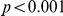 compared to controls. *In vitro* experiments were repeated three times and all assays were performed in duplicate.

We examined release of MIF into the culture media by UROtsa cells in response to different concentrations of thrombin. Figure 3A shows that thrombin stimulation in the range of 10 nM to 200 nM increased MIF released into the culture media in a concentration-dependent manner. The EC50 was determined to be 47.5 nM.

Because a small amount of endotoxin was detected in the thrombin stock (14 EU endotoxin/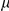
g thrombin), and since endotoxin can elicit MIF release [26], experiments were conducted to rule out that MIF release is due to endotoxin. Stimulating UROtsa cells with 100 nM thrombin significantly increased the amount of MIF released into the culture media when compared to vehicle control (sterile saline: 65.1
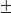
7.4 ng MIF/ml; thrombin: 203.9
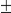
14.6; p
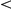
0.001). The effects of treatment with heat-inactivated thrombin (100 nM; 73.7
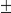
9.9 ng MIF/ml) were not different from control treatment.

We also examined the time course of MIF release into the culture media by UROtsa cells in response to thrombin stimulation (100 nM). Untreated cells (serving as controls) were exposed to media only (no thrombin treatment) for the duration of the experiment (180 min). Media collected from UROtsa cells after thrombin stimulation showed a statistically significant increase in the median amount of MIF at 15 minutes of exposure and reached a maximal effect at 60 minutes of exposure (Fig. 3B). Longer exposure times (90, 120, 180 min) did not produce any further MIF release however MIF amounts remained elevated in the culture media through the observation period (Fig. 3B). In addition, real-time RT-PCR results showed that stimulation with thrombin (100 nM) for 1 hour resulted in an increase in MIF mRNA in UROtsa cells (3.3-fold increase; p
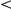
0.05; Table 1) when compared to untreated UROtsa cells.

**Table 1:** Thrombin stimulation upregulates MIF mRNA.

	Treatment	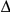 CT^a^	Fold-change^b^
**UROtsa**			
	Untreated	12.49 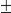 0.20	1
	Thrombin	10.78 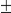 0.62*	3.27
**Rat bladder**			
	Saline	4.84 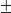 0.16	1
	Thrombin	3.83 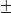 0.12**	2.01
	Lidocaine + Thrombin	4.32 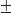 0.31	1.43

a Mean
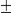
S.E.M.,

b Determined using 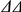
CT method.

*p
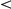
0.05,

**p
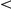
0.01.

### Thrombin evokes MIF release from rat bladder and upregulates bladder MIF mRNA

We also examined the effect of *in vivo* thrombin stimulation on MIF release from the rat bladder. Our results show that treatment with intraluminal thrombin elicited MIF release from the rat bladder (Fig. 3B). In the control group receiving only saline in the intraluminal fluid, the median MIF amount was 1.26+0.4 ng MIF/ml. Intraluminal thrombin (100 nM) for 1 hour increased the median amount of MIF in the intraluminal fluid to 5.68+0.6 ng MIF/ml, a 4.5-fold increase over control (saline treatment) that was statistically significant (Fig. 3B; p
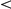
0.01). Concomitant treatment with intraluminal lidocaine (2%) and 100 nM thrombin reduced (compared to thrombin only treatment) the median amount of MIF in the intraluminal fluid to 2.82+4.3 ng MIF/ml, although this amount was still increased compared to control (2.2-fold increase; p
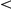
0.05). There was a large variability in the amounts of MIF detected in the lidocaine + thrombin treated group, as reflected in the larger IQR (when compared to the other two groups). In fact, 2/6 rats in this group had intraluminal MIF amounts that were similar to the thrombin only group. Real time RT-PCR showed that thrombin stimulation resulted in upregulation of MIF mRNA in the bladder (2-fold increase compared to saline treatment; p
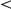
0.001; Dunnet's test) while treatment with lidocaine reduced the effect of thrombin on MIF mRNA upregulation (1.4-fold increase when compared to saline treatment) so that it was not statistically significant from the saline treatment group (Table 1).

## Discussion

The present study shows that: 1) urothelial cells (human and rat) express PAR1 receptors and MIF; 2) Thrombin stimulation of urothelial cells evokes MIF-release *in vitro* and *in vivo* and 3) Thrombin stimulation also induces MIF upregulation in urothelial cells *in vitro* and *in vivo*. These results indicate that activation of PAR1 receptors mediates MIF release from urothelial cell which can then mediate MIF-mediated bladder inflammation, given MIF's pro-inflammatory role in the bladder [6]. Therefore, thrombin-induced MIF release represents another mechanism to initiate MIF release from the urothelium, aside from nerve-mediated release which has already been described [27], [28].

Expression of PAR1 and PAR2 receptors was described for normal human urothelial cells and an urothelial cancer cell line (RT4) [19], [20] and these receptors were shown to be functional since they respond to agonist stimulation [20]. Expression of PAR1-4 receptors has also been reported for an additional human urothelial cancer cell line (J82) however, receptor functionality was not investigated [21]. In the current study we document (using RT-PCR) expression of PAR receptors 1 through 4 in normal transformed human urothelial cells (UROtsa) [29], [30]. In addition, we document that UROtsa cells express MIF and we show, using dual-immunofluorescence that UROtsa cells can express PAR1 and MIF simultaneously. We observed heterogeneity in PAR1 (and MIF) immunostaining in most of these cells with approximately 13% not displaying immunoreactivity for either PAR1 or MIF. Similarly, only approximately 30% of J82 (urothelial cancer cell line) cells were reported to be positive for PAR1 immunostaining [21]. Differences in PAR1 immunostaining may be due to differences in the cell cycle, differences between normal and transformed cell lines or differences in immunostaining protocols and antibodies. In rat urothelium, we also detected MIF and PAR1 immunostaining. PAR1 immunostaining was strongest in basal cells of the urothelium with moderate staining in intermediate cells. No PAR1 immunostaining was observed in umbrella cells. Similarly, basal and intermediate cells also showed MIF immunostaining with umbrella cells showing either slight or no MIF immunostaining. Therefore, the co-existence of PAR1 and MIF is restricted to deeper layers of the rat urothelium.

We also document that thrombin stimulation of urothelial cells, whether *in vitro* (using UROtsa cells) or *in vivo* (using intravesical thrombin application in rats) results in MIF release, and this effect occurs quickly after thrombin application (15 min *in vitro*). Since thrombin contained a small amount of endotoxin and because endotoxin can elicit MIF release [26], it was possible that our thrombin results were due to endotoxin contamination. Heat-inactivated thrombin was ineffective in stimulating MIF release from UROtsa cells which argues against this possibility. Much higher temperatures and longer heating times are needed to abolish the activity of endotoxin [31], [32], therefore it is highly unlikely that our heating conditions affected the activity of the small amount (14 EU/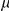
g thrombin) of endotoxin present. Finally, while endotoxin can elicit MIF release it also results in MIF down-regulation [26], [33] which is opposite to the effect of up-regulation seen in our studies. Consequently, our findings indicate that the effects observed from thrombin stimulation are not due to endotoxin. Our results thus confirm earlier findings of thrombin-induced MIF release from human endothelial cells [17] and we extend those results by showing that the same phenomenon occurs *in vivo*. Given that both human and rat urothelial cells were shown to express both MIF and PAR1 we consider it likely that thrombin stimulated PAR1 receptors on rat urothelial cells to elicit MIF release.

Since PAR1 and MIF containing cells in rat urothelium are not located superficially, our findings that intravesical thrombin can induce MIF release from the rat bladder (presumably urothelium) suggest that intravesical thrombin was able to reach those cells to activate PAR1 receptors. Although local thrombin formation during inflammation is likely to occur in the suburothelial compartment and thus stimulate basal and intermediate cells to elicit MIF release, our findings suggest that proteases present in the urine may also be able to activate PAR1 receptors in the urothelium, elicit MIF release and thus contribute to the initiation or maintenance of cystitis. In fact, both mast cell tryptase and neutrophil elastase were documented to be increased in the urine of patients with interstitial cystitis [34], [35], thus raising the possibility that PAR1 receptors could be activated in interstitial cystitis. Activation of PAR1 receptors by neutrophil elastase was reported to induce apoptosis in lung epithelial cells [36] while activation of PAR1 and PAR2 receptors were shown to increase epithelial permeability in intestinal epithelia [37]. Whether these effects can also be seen in urothelial cells, particularly in clinical conditions such as interstitial cystitis, remains to be determined.

Treatment with intravesical PAR1, PAR2 and PAR4 agonists induced inflammation in the mouse bladder [21]. Moreover, PAR1 receptors appear to mediate inflammation caused by a variety of inflammatory stimuli thus emphasizing their central role in the development of cystitis [21]. In addition, activation of PAR1 receptors with specific agonists administered systemically results in plasma extravasation in the rat bladder due to release of SP from terminal afferents [38]. Our current results suggest that one component of PAR1-mediated bladder inflammation may be release of MIF from urothelial cells and MIF upregulation. Bladder/urothelial MIF upregulation during inflammation is a consistent finding regardless of the initiating inflammatory stimulus [6], [8], [39]. We have demonstrated that released MIF is pro-inflammatory since blocking MIF or receptors for MIF reduce morphological and physiological signs of cystitis as well as decrease bladder production of pro-inflammatory cytokines, including MIF [6], [11], [39]. Therefore, thrombin may activate PAR1 receptors in the urothelium to elicit MIF release from urothelial cells and thus continue and/or augment inflammation in the bladder.

**Figure 4:**
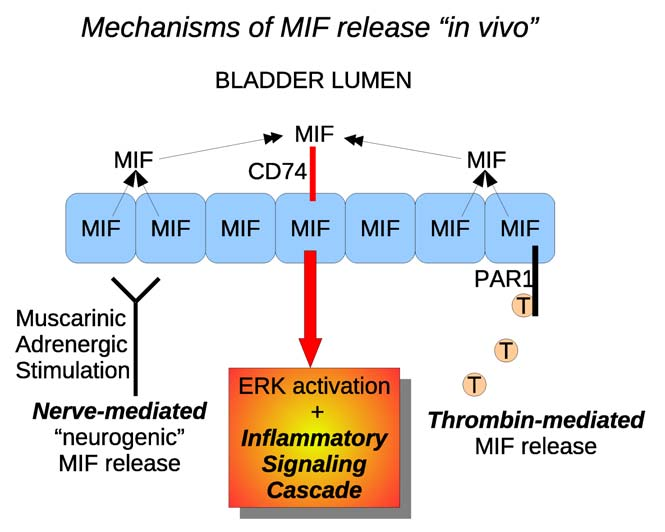
Model of urothelial MIF release mechanisms *in vivo*. Evidence suggests that substance P elicits MIF release in a process that is dependent on bladder afferent and post-ganglionic activation of muscarinic and adrenergic bladder receptors. Since this release appears is initiated by substance P, it is referred to as “neurogenic” MIF release [9], [10], [24]. In addition, findings from the present experiments show that activation of PAR1 receptors in deep layers of the urothelium can also elicit MIF release. Since this effect occurs *in vitro* (UROtsa) cells, this pathway appears to be independent of nerve-activation. However, nerve-activation may still contribute, since intravesical lidocaine (to stop all bladder nerve activity) reduced such release *in vivo*. This alternate pathway for MIF release may be activated by thrombin generated locally as part of the inflammation process or may be activated by proteases found in the urine. Activation of either or both pathways results in MIF release from urothelial cells, MIF binding to cell-surface CD74 in urothelial cells and activating extracellular regulated kinase (ERK) signaling and a pro-inflammatory cytokine cascade. Thus, by standing upstream of such a pro-inflammatory cytokine cascade, MIF maintains or augments bladder inflammation.

We showed previously that substance P elicits MIF release from the bladder in general, and urothelium in particular, dependent on bladder nerve activation [9], [10], [24]. Therefore, it is possible that intravesical thrombin may also be functioning in a similar manner to elicit MIF release. Our results with intravesical lidocaine + thrombin treatment indicates that a considerable portion of MIF released is due to non-neurogenic mechanisms. We consider it a likely explanation that direct stimulation of MIF and PAR1 containing cells in the urothelium is involved in thrombin-stimulated MIF release in the rat bladder. In fact, our earlier results investigating MIF release during neurogenic inflammation (Substance P-evoked) showed that intravesical lidocaine abolished MIF release in that model [27], and thus are different from findings in the present study. These observations suggest that there may be two components to MIF release, one a neurogenic component involving SP release and a direct mechanism involving PAR1 stimulation of urothelial cells (Fig. 4). Blocking activation of PAR1 receptors, blocking MIF or receptors for MIF should thus lead to decreased bladder inflammation.

## Materials and Methods

### Ethics Statement

Animal experiments were approved by the Bay Pines VA Healthcare System Institutional Animal Care and Use Committee (approval no. 2636) and conformed to NIH guidelines for animal experimentation.

### *In vitro* experiments

Normal immortalized human urothelial cells (UROtsa; derived from normal urothelium lining the ureter and transformed using simian virus 40 [29], [30]; a kind gift of Scott H Garrett) were routinely cultured in plastic flasks in Dulbecco's modified Eagle's medium (DMEM) with Glutamax supplemented with 10% fetal bovine serum (FBS) (Invitrogen Life Technologies, Carlsbad, CA) at 37 C in 5% 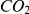; 95% air environment. Cells were plated in 24 well plates at a density of 6×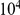 cells/ml and incubated overnight. Growth medium was removed and DMEM with 0.1% bovine serum albumin added. Cells were incubated for 1 hour at 37

C and then exposed to thrombin at different concentrations (in sterile water; Sigma-Aldrich; St. Louis, MO) for 1 hr and culture medium collected. Thrombin was tested for the presence of endotoxin using a chromogenic *Limulus* Amebocyte lysate assay (Genscript; Piscataway, NJ; #L00350C) according to the manufacturer's protocol. In order to establish that the effects of thrombin were due to thrombin and not to endotoxin, control experiments examined the effects of UROtsa stimulation with thrombin vs heat-inactivated thrombin (60

C; 10 min).

In a separate set of experiments, UROtsa cells were plated and incubated as above, growth medium removed and replaced with DMEM with 0.1% bovine serum albumin and were incubated for 1 hour at 37

C and then exposed to thrombin (100 nM) for the following time intervals: 0, 15, 60, 90, 120, 180 min. Culture medium from untreated cells was collected at 180 min and served as control.

### *In vivo* experiments

Male rats (250–275 g; Sprague-Dawley; Harlan; Indianapolis, IN) were anesthetized with halothane and placed on a heating pad. A ventral abdominal incision exposed the bladder and ureters. Both ureters were cut and allowed to drain to gauze while urine was removed from the bladder using a 30 ga needle and a hypodermic syringe. Rats were divided into three groups (n = 6/group) and 0.3 ml of either 1) sterile saline (vehicle control); 2) 100 nM thrombin; or 3) 2% lidocaine +100 nM thrombin were injected into the bladder lumen. After 1 hour the intraluminal fluid was collected and stored at −80

C until MIF ELISA analysis (see below). Bladders were removed, sectioned in half longitudinally and stored at −80

C for RNA extraction and placed in formaldehyde for immunohistochemistry and the rats were euthanized with an overdose of halothane and a thoracotomy. Because formaldehyde fixation proved deleterious to PAR1 immunostaining (see Results below), the bladder of one additional rat was collected under halothane anesthesia, frozen in liquid nitrogen and stored for immunohistochemistry (described below).

### RT-PCR and Western blotting

RNA was extracted from UROtsa cells and bladder tissues using TRIzol reagent (Invitrogen). One 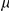
g of the resulting total RNA was reverse transcribed to cDNA using Avian Myeloblastosis Virus (AMV) reverse transcriptase and random primers (Promega; Madison, WI).

To determine the expression of PAR 1–4 receptors in UROtsa cells, approximately 2×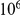 subconfluent cells were plated in six well plates and incubated overnight in DMEM supplemented with 10% FBS under standard conditions and cells harvested by the direct addition of TRIzol reagent. cDNA for PARs 1, 2, 3 and 4 were amplified by endpoint PCR with specific primers and RT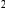 SYBR green PCR master mix (Qiagen; Valencia, CA) using a three step cycling program: 95

C, 15 sec; 55

C, 40 sec; 72

C, 30 sec for 40 cycles on a C1000 thermocycler (BioRad, Hercules, CA). 20 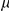
l of the resultant PCR reaction was separated on a 4% E-gel (Invitrogen) and the image captured using Kodak Image Station (Kodak, Rochester, NY).

Thrombin induced upregulation of MIF expression in UROtsa cells was determined by real-time PCR (Opticon, BioRad). Total RNA from thrombin treated cells (as described above, *in vitro* experiments) was isolated by the direct addition of TRIzol reagent (Invitrogen). cDNA was diluted 1∶1 with nuclease-free water and 5 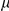
l of the diluted cDNA was amplified using RT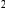 SYBR green PCR mix and human MIF specific primers (Qiagen) using the following cycling program: 95

C, 10 min followed by 40 cycles of (95

C, 15 sec; 55

C, 30–40 sec, and 72

C, 30 sec). The 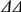
CT method was utilized to determine fold changes using 18S rRNA as the housekeeping gene. Data are from triplicate wells. Thrombin induced upregulation of MIF expression in rat bladder tissue was determined by real time PCR. One-quarter of the snap frozen bladder was homogenized in TRIzol reagent. cDNA was diluted 1∶1 with nuclease-free water and 5 ul of the diluted cDNA was amplified using RT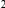 SYBR green PCR mix and rat MIF specific primers (Qiagen) as described for UROtsa cells.

For protein extraction, cells were directly lysed by the addition of 1X NuPAGE sample buffer (Invitrogen). The DNA in the resulting cell lysates was sheared by passing through a 25 ga needle. Equal volumes of the lysates were separated by 4–12% Bis-Tris SDS-PAGE (Invitrogen) and transferred to a polyvinylidene fluoride membrane. Blots were blocked with Odyssey blocking buffer (Li COR; Lincoln, NE) for 1 h at 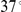
C and incubated overnight with affinity purified goat anti-human MIF polyclonal antibody (R&D Systems; Minneapolis, MN; AF-289-PB; 1/1000 dilution) at 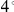
C, followed by incubation with an anti-goat secondary antibody labeled with infrared dye 800CW (Li COR). Individual protein bands were visualized using an Odyssey imager (LiCOR).

### MIF ELISA

MIF amounts in the culture media of UROtsa cells exposed to thrombin were determined using a commercially available ELISA to detect human MIF, according to the manufacturer's protocol (R&D Systems). MIF amounts in rat intraluminal fluid were detected using a validated, custom ELISA protocol for detecting rat and mouse MIF developed in our laboratory and described previously [6]. *In vitro* experiments were repeated three times and all assays were performed in duplicate.

### MIF and PAR1 Immunohistochemistry

UROtsa cells were grown in chamber slides, briefly rinsed in cold (4

C) phosphate-buffered saline (PBS) and then fixed either in frozen in cold (4

C) 4% paraformaldehyde or cold (−20

C) solution of 3∶1 acetone:PBS for 10 min. Slides were rinsed with PBS and exposed to primary antibodies as follows: 1) Goat anti-PAR1 (R&D Systems; 1∶100) or Goat anti-PAR1 (BD Bioscience; San Jose, CA; 1∶50) or Mouse anti-PAR1 (Santacruz, Santa Cruz, CA; 1∶100) 2) Rabbit anti-MIF (Abcam; Cambridge, MA; 1∶100) in PBS overnight at 4

C. and visualized using AlexaFluor (Invitrogen) conjugated secondary antibodies (1∶200; Donkey anti-goat AlexaFluor 488; Donkey anti-rabbit AlexaFluor 555). Paraffin (4 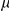
m) or sections of formaldehyde-fixed bladders and frozen (14 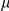
m) sections of intact (fresh-frozen) bladder were collected. Intact bladder sections were also fixed in cold (−20

C) acetone for 10 min, allowed to air dry for 1 hr at room temperature and exposed to primary antibodies as follows: 1) Goat anti-PAR1 (1∶50; R&D Systems); 2) Rabbit anti-MIF (1∶400; Torrey Pines; East Orange, NJ) in PBS overnight. Primary antisera were then detected using secondary antibodies conjugated to Alexafluor. Slides were coverslipped with Prolong Gold (Invitrogen; containing DAPI as a nuclear stain) and examined on an Olympus FV100D laser confocal microscope. Images of individual dye immunostaining and overlays of both dyes were obtained using ImageJ [40].

### Statistical Analysis

For the effect of different concentrations of thrombin on UROtsa MIF release, a concentration-response curve was fitted using log-logistic regression with 4-point parameter estimation (using R [41] and the drc package [42] and the ED50 was calculated. Differences in MIF release by UROtsa cells that were treated with the addition of sterile saline (control), thrombin (100 nM) or heat-inactivated thrombin (100 nM) are presented as Mean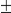 S.E.M and were analyzed using ANOVA, followed by Dunnet's tests (if ANOVA reached significance). The remaining MIF ELISA results are presented as Median + Interquartile Range (IQR) and were analyzed using Kruskal-Wallis ANOVA followed by post-hoc tests with comparisons to the control group using statistical software program (R; [41] and the pgirmess package [43]). Real time RT-PCR results (changes in 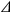
CT) were analyzed by Student's t-test in the case of UROtsa cells comparing thrombin stimulation to untreated cells and by ANOVA using R [41] followed by Dunnet's tests (if ANOVA reached significance) in the case of rat experiments, using the saline treatment group as control. A p value 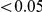 was considered statistically significant.
